# Approaches to Determine and Manage Sexual Consent Abilities for People With Cognitive Disabilities: Systematic Review

**DOI:** 10.2196/28137

**Published:** 2022-02-04

**Authors:** Shaniff Esmail, Brendan Concannon

**Affiliations:** 1 Department of Occupational Therapy University of Alberta Edmonton, AB Canada; 2 Faculty of Rehabilitation Medicine University of Alberta Edmonton, AB Canada

**Keywords:** sexual consent, capacity, disability, sexual expression, dementia, ethics, long-term care

## Abstract

**Background:**

This review focused on how sexual consent ability was determined, managed, and enhanced in people with cognitive disabilities, with the aim of better understanding the recurring themes influencing the design and implementation of these approaches. If a person’s consensual ability becomes compromised, owing to either an early or late-onset cognitive disability, the formal systems involved must establish plans to balance the individual’s rights and restrictions on sexual expression. This review identified these plans, focusing on how they promoted the intimacy rights of the individual.

**Objective:**

This study aims to identify approaches that determine sexual consent ability in people with cognitive disabilities, identify the means of managing and enhancing sexual consent ability in people with cognitive disabilities, and note the recurring themes that influence how these approaches and management systems are designed and implemented.

**Methods:**

A systematic literature review was performed using EBSCOhost (Social Gerontology, CINAHL Plus, MEDLINE, and SocINDEX), Embase, PsyInfo, and Scopus to locate reports on terms expanded on sexual consent and cognitive disability.

**Results:**

In all, 47 articles were identified, featuring assessment practices, legal case studies, and clinical standards for managing sexual consent capacity in people with cognitive disabilities. A total of 8 studies (5/8, 63% qualitative and 3/8, 38% quantitative) were included out of the 47 articles identified. Approaches for determining sexual consent included functional capacity and person-centered, integrated, and contextual approaches. Management of sexual consent ability included education, attitude, and advanced directives and support networks. The recurring themes that influenced these approaches included the 3 legal criteria of consent, American Bar Association and American Psychological Association Model, Lichtenberg and Strzepek Instrument, Ames and Samowitz Instrument, Lyden approach, Mental Capacity Act of 2005, and Vancouver Coastal Health Authority of 2009.

**Conclusions:**

Determining sexual consent takes a holistic approach, with individuals judged in terms of their adaptive abilities, capacities, and human rights. The attitudes of those using this holistic approach need to be balanced; otherwise, the sexual rights of assessed people could be moved either in favor or against them. The ideal outcome, after person-centered considerations of those living with cognitive disabilities includes the people themselves being involved in the process of personalizing these approaches used to facilitate healthy intimate relationships.

## Introduction

### Background

#### Defining Cognitive Disability, Sexuality, and Consent

Cognitive disabilities are defined as long-term mental impairments, including those of intellectual and developmental order. The terms *cognitive* and *intellectual* are often used interchangeably, with intellectual disabilities defined as a limitation in academic functioning based on standardized intelligence tests and IQs, associated with IQ scores <75; limitations in learning behavior to one or all three skill types—conceptual skills, social skills, and practical skills; and manifestation of the disability before the age of 18 years [1,2]. Noncognitive mental conditions, including psychiatric and psychosocial conditions, are associated with anxiety, mood, and personality disorders.

Sexuality is a holistic concept encompassing sex, gender identity, orientation, eroticism, intimacy, and reproduction [3]. It is influenced by physical, psychological, social, financial, cultural, legal, historical, and spiritual factors [3]. Sexuality includes sexual expression. The central debate surrounding the rights of people with cognitive disabilities, who wish to express their sexuality, may affect the balance between harm reduction and free sexual expression [4]. If a person living with a cognitive disability is proven to have a reduction in consensual capacity, the protection versus empowerment paradox may begin to emerge [5,6]. The legal, clinical, or ethical system needs to find a balance between the 2 competing interests; protect the person from sexual abuse, by restricting their sexual expression; or allow them to express their sexuality, but in limited capacity as a safety measure [5]. An important factor affecting the resolution of the protection versus empowerment paradox is the degree to which an individual can demonstrate their capacity to consent in a sexual relationship.

In the United States, the legal definition of consent is rooted in the 3 legal criteria of consent as reported by Stavis [7] in 1991. A placeholder definition of consent requires that a person communicates a “knowing, intelligent, and voluntary agreement to engage in a given activity” [8,9]. Assessments that measure consensual ability for a sexual relationship are often based on a person being able to satisfy all three of the following criteria [3,7,10,11]:

1. Knowledge—recognition of the other person in the relationship, including who, what, where, and when and safety aspects of the sexual activity in question, such as the ability to identify body parts.
2. Intelligence—also known as rationality or understanding, which includes awareness of potential risks (pros and cons) of sexual engagement, appropriateness, consequences, correct familiarity of partner identity, and the ability to discriminate among fantasy, reality, lies, and truth.
3. Voluntariness—decisional capability to engage or refrain from sexual activity and the ability to take self-protective measures against abuse and exploitation or other unwanted advances. This includes the ability to say “no,” either verbally or nonverbally and the ability to remove oneself from the situation when either they or their partner indicates stopping sexual behavior.

These 3 legal criteria of consent are quite controversial, because thresholds vary from basic to complex levels of acceptability, depending on differences in state laws [12]. People with cognitive disabilities may be unable to fulfill requirements in one region of the United States, yet their ability to demonstrate consent could be acceptable in another region. Outside of the United States, consent definitions may differ among countries such as the United Kingdom, Australia, Ireland, and Canada. In Canada, criminal offenses, including those regarding consent, are governed by the *Criminal Code* [13]. Criminal law powers are under the exclusive jurisdiction of the federal government; however, the fulfillment of these laws is often handled by provincial regulations (eg, dealing with sexual assault on university campuses) [14]. The *Criminal Code* defines consent with a capacity element, which checks if an individual understands the nature of sexual activity, identity of those involved, and their ability to communicate the choice itself [13].

The capability of people to satisfy sexual consent criteria is often determined by either medical professionals or neuropsychological experts in the judicial system. Common paradoxes have emerged, owing to the philosophical arguments surrounding people’s ability to give consent. These paradoxes include whether people can demonstrate rudimentary versus contextual understanding of the sexual relationship [15] and the degree of flexible versus inflexible behavioral allowances in such a relationship [16]. Power dynamics, regarding those who are legally able to discern a person’s consensual ability, are met with arguments of feminism, ableism, and disability rights movements [17,18]. The 3 legal criteria of consent have been accused of failing to consider an individual’s values, culture, and life history [10,19]. Outside the United States, the United Kingdom and Wales follow the Mental Capacity Act 2005, which has its own criticisms. Although the Mental Capacity Act has similar themes to the 3 legal criteria of consent, it too is criticized because “its best-interests approach is paternalistic” [20]. The Mental Capacity Act does not exactly guarantee the rights of an individual, instead only working *if practicable to do so* [21]. Some experts argue that an individual’s sexual preference is a form of personal expression, not always systematic or organized, including the weighing of risks and benefits, unlike the 3 legal criteria of consent by the medical and judicial systems in the United States [10,19]. Consent capacity is considered a state instead of a trait, meaning it is expected to change over time [11], and it must be determined in the present moment: not a decision made ahead of time [22]. Assessments of a person’s ability to demonstrate capacity in 1 or all 3 prongs of consent can be determined by questionnaires such as the Mini-Mental Status Exam (MMSE) to determine rationality or the Tool for the Assessment of Levels of Knowledge Sexuality and Consent [11]. However, the use of these assessments is controversial because of the following:

1. Rudimentary requirements that check for consent capacity may fail to understand the contextual reason to *why* a person with a cognitive disability may wish to consent to sex [18].
2. Complex knowledge of consent may have assessments and protocols that are too difficult for even the general population to pass [23].
3. The *Ice Cream Reference*—a person with a cognitive disability is expected to follow a rigid medicalized or judicial process in order demonstrate their consent capacity; however, decisions about sexual relationships are arguably more related to selecting a flavor of ice cream than, say, a life-or-death surgical treatment [24].
4. Assessments of consent capacity often place the burden of proof on the person with a cognitive disability rather than putting the onus on others to prove otherwise. Having individuals provide predetermined comfort with various levels of intimacy carries an unfair standard, because even the general population may not know what levels of intimacy they are comfortable with before engaging in such behaviors [4].

There is no clear definition, criteria, or standard for determining a person’s sexual consent capacity [5,9].

#### Human Rights and Sexual Abuse

In the late 1960s, the United States Supreme Court declared constitutional rights for people with cognitive disabilities, who were cared for under the powers of the state governments. These constitutional rights were created to protect vulnerable people from harm related to sexual exploitation and abuse, while also upholding their rights to sexual expression. These rights include several categories, including those related to family matters [7] and sexual self-determination [4]. Although it may seem obvious that people have default rights to privacy, sexuality education, and freedom of choice for sexual expression [3,25], these rights can be restricted by either informal or formal control systems. Informal control systems such as civil liability [26], immutable family policy [22], and residential policy [4,27,28] may interfere with a resident’s rights to sexual expression, whereas restrictions from formal control systems may be decided by clinical, ethical, and legal issues [26]. The central reason to why these systems may restrict a person’s right to sexual expression is based on the theme of consent [1,15,18,29]. New York Penal Law Section 130 states that a lack of consent is an element of every conceivable sexual offense, as written in the article, and adults in a sexual relationship must all be consenting [30]. Since 2012, the United Nations has moved toward an equalization stance on the sexual rights of people living with cognitive disabilities. Article 12 of the United Nations Convention on the Rights of Persons with Disabilities (UNCRPD) states that people with cognitive disabilities reserve equal rights to legal capacity as all other people within all aspects of their lives, including the rights to intimate relationships [31]. Canada has accepted and ratified Article 12; however, with reservation, resulting in continued use of substitute decision makers (SDMs) to assist those living with cognitive disabilities in Canada [32]. The United States has been hesitant to fully accept and ratify the UNCRPD statement on the rights of people with disabilities [33]. It is important for legal systems to establish the components of human rights, because this increases the awareness of any potential violations.

Sexual abuse occurs when one person forcefully or covertly performs nonconsensual sexual acts, including touching, kissing, oral sex, and anal or vaginal intercourse [3,34]. Sexual abuse may also involve threatening, coercing, tricking, or manipulating another person into unwanted sexual contact or into such contact in which the other person does not have the capacity to consent [11]. According to the *Criminal Code* of Canada*,* an alleged case of sexual assault requires a juridical system to check if people take reasonable steps to ascertain consent [13]. Other elements are also checked for, such as the presence of physical force, threats, underaged individuals, fraud, sexual intentions and motivations, recklessness, incapacitation, and chemical impairment and those in positions of authority or trust [13].

People with cognitive disabilities have a greater risk of being sexually abused [3,25]. Although the statistics on sexual abuse are difficult to determine, one report predicts that 39% to 68% of female children and 16% to 30% of male children with cognitive disabilities will be sexually abused before they are aged 18 years in North America [35,36]. After interviewing over 40,000 abused victims in Israel, including children up to the age of 14 years and ranging from minor to severe cognitive disabilities, the incidence of sexual abuse was found to be consistent with the previously stated percentages [37]. A survey of over 5000 adult women living with cognitive disabilities in North Carolina reported that 48% of sexual assaults were committed by people who were currently or previously in an intimate relationship [38]. A 15-year longitudinal study in Ireland determined the following statistics after 118 proven and confirmed episodes of sexual abuse [39]:

1. Most of the perpetrators were men.
2. A percentage (n=66) of the perpetrators had a cognitive disability.
3. In all, 24% (n=28) of the perpetrators were relatives.
4. In all, 9% (n=11) of the perpetrators were agency staff members.
5. In all, 8% (n=9) of the perpetrators were familiar people.
6. The remaining perpetrators were either volunteers, strangers, or unknown.

#### Society Attitudes on the Sexuality of People With Cognitive Disabilities

The *zeitgeist* to uphold and safeguard the sexual rights of persons with cognitive disabilities may differ from past ideals, which were weighted toward the protective sides of the protection versus empowerment paradox [5,40]. Societal views on the amount and types of sexual expression that people with cognitive disabilities were expected to experience were driven by moral aesthetics, which are beliefs and morals that affect the general public’s preference to accept certain behaviors while rejecting others. Thus, people with cognitive disabilities were historically denied the right to express their sexuality, because society may have considered them to be the following:

1. Hypersexual—*oversexed* people who were often seen as a threat to the gene pool and general public, owing to their excessive sexual behavior [8,11]. These people may have been identified as having *super human strength* sex drives [18]. Reported cases of older adults living with dementia may repeatedly approach partners for sex, after forgetting they had sex earlier [41]. An emerging tendency toward public masturbation is a potential problem for older adults living with dementia [41]. Child masturbation can also be a common form of childhood sexual behavior, which is considered *developmentally normal*, unless inappropriate owing to public occurrence, excessiveness, or when the behavior causes injury [42-44].
2. Asexual—*eternal children* were often seen as potential sexual victims who were deemed to have a major difference in their chronological and mental ages [45] and assumed to not necessarily want sexual relationships or need sexuality education, because it may incite increased interest in the activity or the risk of abuse [3,18,46]. Therefore, some policies thought better to keep *Pandora’s box closed* to reduce these risks, which actually increased the vulnerability of these people, due to the lack of education about those who might exploit them in the first place [25,47]. *Pillow Angels* are defined as people with cognitive disabilities who were thought to be incapable, or should be made incapable, of becoming adults and were removed from sexual relationships to be spared the dangers of sexuality, such as pregnancy and sexual exploitation [48,49].
3. Deviant—in the last 20 years of research on the well-being of people who are lesbian, gay, bisexual, transgender, or queer (LGBTQ), there is evidence of victimization among such sexual and gender minorities in both youth and adults [50]. Although initial perceptions of the North American society show a more open and tolerant view of the LGBTQ community, victimization rates and disparities have worsened since the 1990s [50-53]. LGBTQ older adults, living with cognitive disabilities, are often met with *pervasive stigma* by staff in long-term care (LTC) facilities, who have reported to feel disgust or panic, sometimes resorting to denial of such residents’ sexuality [22,29,54,55].

The moral aesthetic to control how people with cognitive disabilities express their sexuality are bound to clinical, ethical, and legal issues [26]. There is a potential overlap among these issues.

Social acceptability struggles to find a balance between sexual acts that are safe versus unsafe, normal versus deviant, and legal versus illegal and what role sexual functioning has in the first place [34].

Clinical policies in a LTC facility could be undeveloped or inconsistent with those living with cognitive disabilities and their sexual expression, resulting in the facility facing repercussions if sexual expression is allowed to continue [26,56]. LTC facilities for people with cognitive disabilities default to a *protective care paradigm*, with staff and family members restricting such residents from sexually expressing their behaviors to reduce the risk of potential sexual abuse [29]. The result is that such residents may resort to *opportunistic moments of privacy to act on their sexual desire* [48,57], which may lead to unsafe sexual behaviors [56,58]. Alternatively, they could be affected by iatrogenic loneliness, which is a type of loneliness created by extensive long-term residence policies that prevent them from having privacy and intimacy, resulting in feelings of frustration and unhappiness [28,59]. LTC staff views on sexual expression differ according to the experience levels of staff members, with frontline staff being more accepting of such behaviors than the managers; however, gay residents are more likely to be restricted to such behaviors in general [29]. Ethical views, independent of those with cognitive disabilities, include moral and religious beliefs that others enforce regarding sexual behavior [26]. In theory, a LTC facility must support such a resident’s rights to sexual autonomy; however, this obligation is abandoned once the administration, facility staff, or individual’s family members oppose the behavior [4,22]. Fear of legal repercussions and public ridicule are potential reasons why such people’s sexual interests are downplayed or avoided by family, caregivers, or long-term facility staff [23]. Overall, the community is capable of supporting the sexuality of people with cognitive disabilities, upholding attitudes of community inclusion and opportunity; however, personal belief systems are affected by societal attitudes and are what prevent caregivers from providing experiential guidance [34,60]. Negative attitudes, such as the eugenics movement, were perhaps too difficult for North American society to discard entirely [48,61]. Thus, society *morphed* them to the new era, resulting in *new-genics* or *neogenics* [62]. The intention of determining a person’s capacity to consent to sex has remained a *plague* in the societal attitude to desexualize people with cognitive disabilities, under the moral esthetic to either protect such individuals from themselves or to protect the world from them [63].

#### Sterilization and Eugenics

Sterilization is the process of inhibiting a person’s reproductive ability. It inflicts physical and moral injuries to those who do not consent to it [21,64]. The eugenics movement of the late 19th century led to an increase in nonconsensual sterilization practices, sometimes with the use of deception [21]. Eugenicists believed that the human race could be improved by practicing either positive or negative eugenics, which either encouraged the selective breeding of those with desirable traits or the prevention of *defectives* from having offspring [65]. In the early 1960s, 28 US states had sterilization protocols, some made compulsory and executed upon people with cognitive disabilities, without their consent [3].

The justification for sterilization was often influenced by the eugenics movement, which believed that *feebleminded* people would reduce the overall intelligence of the population, especially if they were allowed to reproduce [21]. It was believed that people with cognitive disabilities would threaten the *heritage of intelligence* [40,66,67]. In actuality, the eugenics idea to use selective breeding to control for inherited psychological traits was proven to be false [21,68]. Feeblemindedness was previously used as a *conveniently vague grouping*, used to classify those who were outside the obvious diagnostic labels such as schizophrenia [21]. Some countries sterilized those diagnosed as *feebleminded*; however, it was later realized that many of these people were actually affected by a lack of education [21]. In a report from India that surveyed nearly 20,000 women, higher levels of education increased the likelihood of modern contraceptive use over sterilization; however, the degree of cultural, socioeconomic, educational, and accessibility to modern contraceptives had a profound effect on choice [69]. In India, sterilization was more common in women living in low socioeconomic classes, especially in socially disadvantaged women with low education levels [69]. Sterilization was sometimes ordered because of the protection versus empowerment paradox. Some systems believed that women with cognitive disabilities would fail to provide adequate care for any children they would have; thus, these potential mothers were prevented from leaving their institutions unless they agreed to become sterilized [70]. With the exception of reducing pregnancy and reducing bodily fluids, sterilization of people with cognitive disabilities was found to be ineffective in achieving any of its goals [71,72]. There is limited evidence available to support sterilization for the management of menstruation, with some experts agreeing that cases involving clinical control of menstrual bleeding are better handled by long-term contraceptive injections [73].

There have been major changes in legislation regarding the practice of nontherapeutic sterilization [21]. In Canada, a major case occurred in 1986. This *E (Mrs) versus Eve* case argued that court-ordered sterilization of people, living with cognitive disabilities, would be an infraction against their rights. The mother requested sterilization of her daughter, Eve, aged 24 years, to avoid the risk of pregnancy. After a contentious appeal, the request was denied. The Supreme Court of Canada ruled in favor of Eve, due to a lack of evidence suggesting that forgoing sterilization would have a detrimental effect on physical or mental health in Eve [74]. The choice to allow nontherapeutic sterilization of people with cognitive disabilities, in which a procedure would leave a person sterile despite having no life-threatening condition to begin with, was deemed a choice the courts could not safely exercise [21,74]. Later reports have claimed that some countries have implemented human rights protection to prevent nonconsensual sterilization practices; however, some countries have no such safeguards in place [21]. The following references contain additional information pertaining to the statistics and country policy on sterilization: Stein and Tepper [3], Rowlands and Amy [21], Braun et al [64], Tilley et al [70], Shea and Kevles [75], and Park and Radford [76].

#### Benefits of Healthy Sexual Expression

There are psychological and physical benefits of safe sexual expression. Improved self-esteem, cognitive functioning, social relationships, mood, and feelings of independence have been reported as potential benefits [77,78]. Sexual expression may reduce the risk of cancer and cardiovascular disease [77]. There are associations between sexual expression and weight loss, reduced risk of heart disease and stroke, and bolstered immune systems [79]. For older adults with cognitive disabilities, sexual expression has been reported to reduce sensitivity to pain, improve cardiovascular health, reduce the likelihood of depression and loneliness, and improve overall well-being [80]. Sexuality is *central to an individual’s health and well-being* [4].

### Aim of This Review

This review aims to uncover the used approaches of clinical, legal, and residential systems to determine and manage the sexual consent abilities of people with cognitive disabilities. Recurring themes influencing the shape of these approaches were also identified. Specific audiences for this review include human ecologists, sexuality experts and therapists, forensic neuropsychologists, occupational therapists, sexual educators, health care professionals, service providers, and caregivers.

### Objectives

The objectives of this review are as follows:

1. Identify approaches used to determine sexual consent ability in people with cognitive disabilities.
2. Identify means of managing and enhancing sexual consent ability in people with cognitive disabilities.
3. Note the recurring themes affecting how such approaches and management systems are designed and implemented.

## Methods

### Research Question

This report presents a systematic review of the literature, based on consultation with human ecology and rehabilitation medicine experts, to create the following research question: What are the approaches for determining, managing, and improving sexual consent ability in people with cognitive disabilities?

### Search Strategy

After discussing the research question with a university librarian, the following bibliographic databases were searched: EBSCOhost (abstracts in Social Gerontology, CINAHL Plus, MEDLINE, and SocINDEX), Embase, PsyInfo, and Scopus. The search strategy included a combination of subject headings and keywords to combine the concepts of consent in sexuality and cognitive disability. Textbox 1 lists the inclusion and exclusion standards for each article. The full search strategy is provided in Multimedia Appendix 1.

A total of 2 researchers performed the screening process for each article (BJC and recruited researcher, Lyndsay Pinder). Differences among the researchers in terms of accepted and rejected articles were resolved through discussion. All articles indicating topics of sexuality and consent within their titles or abstracts were reserved to complete the first pass of the search process (BJC and Lyndsay Pinder). For the second pass, all reserved reports from the first pass had their full texts screened to confirm the context of the subject (BJC, SE, and Lyndsay Pinder). The methodological quality of the reports featuring experimentation was not formally assessed. There were no data limits.

**Search criteria and terms.**

**Inclusion criteria**

- Article stated a topic, discussion, or approach to determine the consent capacity of people with cognitive disabilities.
- All articles featuring qualitative, quantitative, legal, descriptive, and review reports were accepted.
- Reports were accepted in all languages and in article, dissertation thesis, review, or book format.

**Exclusion criteria**

- Topic was about physical disability or did not indicate a potential compromise in a person’s consensual ability.
- The article briefly mentioned
  consent to sexuality
  or a similar phrase; however, further details were not provided.
- Conference papers, public opinions and non–peer-reviewed articles.

**Search terms used**

- ([sex* or intima*] adj10 [consent or consensual]) AND ([(intellectual* or mental* or cognitive*) adj4 (impair* or disab* or deficit*)] or long term care or longterm-care or nursing home* or alzheimer* or dementia or autis* or Down* Syndrome).
- These terms were entered into the databases mapped to the following fields: title, abstract, subject heading word, and keyword heading word.

### Search Results

**Figure 1:**
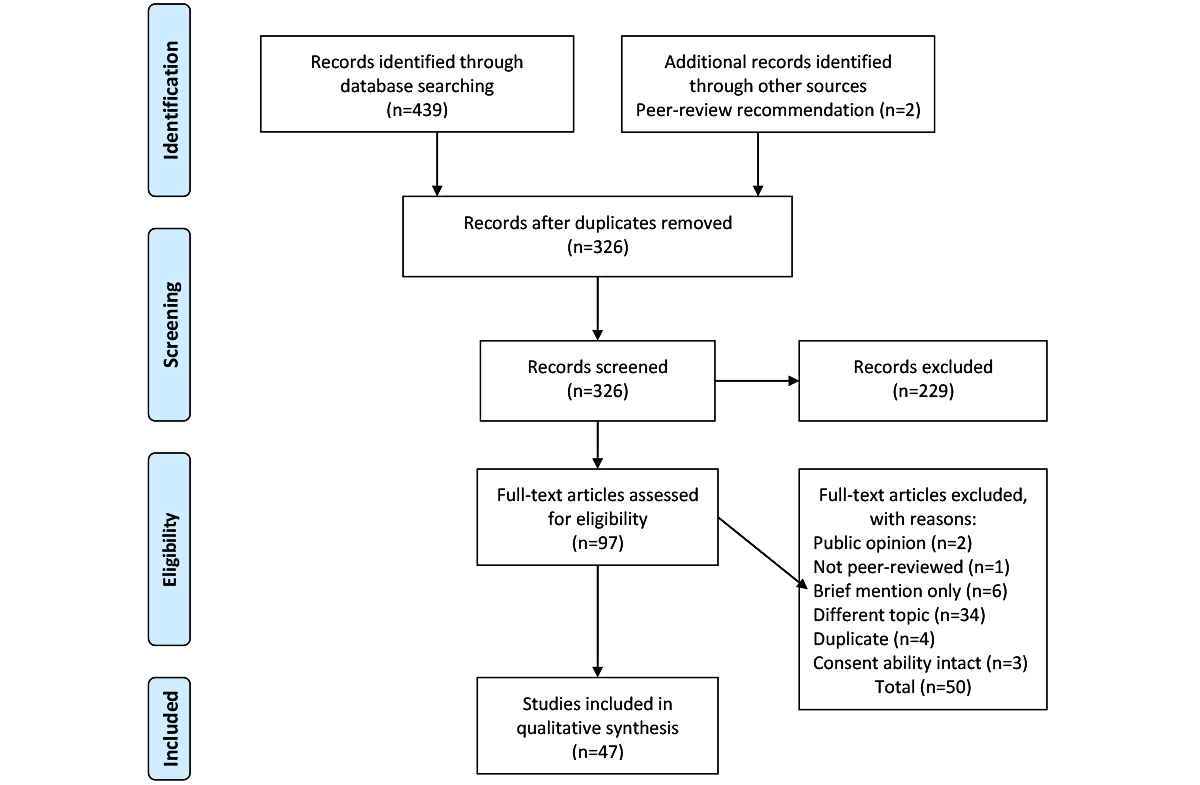
PRISMA (Preferred Reporting Items for Systematic Reviews and Meta-Analyses) flow diagram of search results [82].

The search resulted in 439 articles being identified, of which 2 (0.5%) articles were recommended for inclusion in the peer-review process [12,81]. After the first pass, 22.1% (97/439) articles remained after the titles and abstracts were screened, and 26.2% (115/439) duplicates were removed. During the second screening pass, the full texts of 97 articles were screened to confirm eligibility (Figure 1). This resulted in a net total of 10.7% (47/439) articles being included in this review.

## Results

### Overview

The 47 reports included in this review featured assessment practices, legal case studies, and clinical standards for managing sexual consent capacity in people with cognitive disabilities. Most reports were in the form of expert opinions (36/47, 77%). There were 8 studies (5/8, 63% qualitative and 3/8, 38% quantitative) included in this study. The qualitative studies included the following:

1. A survey with vignettes to check the ability of residential facility staff to properly identify safe or unsafe sexual behaviors (nonconsensual sexual behavior) in people with cognitive disabilities and respond accordingly [34].
2. A survey of members of the American Psychological Association to determine which criteria were considered the most important when determining sexual consent capacity in people with cognitive disabilities [9].
3. A survey to determine factors that increase the risk of SDMs to decide an *all-or-none* outcome for a person’s sexual autonomy [83].
4. Semistructured interviews in residential mental health treatment facilities to determine what conceptualizes consent to sexual expression from the point of view of administrators, clinical staff, and former clients [84].
5. Semistructured interviews with directors of nursing to identify challenges in managing sexual expression [85].

Quantitative studies focused on educational interventions for the improvement of sexual consent ability in people with cognitive disabilities [40,86]; 1 study performed a validity measure to compare neuropsychological tests with the Sexual Consent and Education Assessment [45]. A total of 4 reports were dedicated to introducing a theme, which would later influence the approaches used to manage or enhance the sexual consent capacity of people with cognitive disabilities. Table 1 provides a summary of each theme, and Table 2 provides a brief description of each approach. The 2 approaches for determining or managing consent were peripheral to these themes [12,81]. For more information about the research studies, refer to Table 3.

**Table 1:** Themes affecting the approaches for determining sexual consent capacity in people with cognitive disabilities.

Theme	Key components	References
Lichtenberg and Strzepek Instrument	Interdisciplinary characteristics. Client is assessed (MMSE^a^), followed by a same-sex interview to determine these three main criteria: Awareness of the relationship—patient aware of intent, partner identity, and intimacy comfort level. Ability to avoid exploitation—patient behavior consistent with former beliefs and able to say no. Awareness of potential risks—consequences of relationship and awareness of relationship duration. Interview relayed to interdisciplinary team (nurses, occupational therapists, psychiatrists, etc).	[22,87-89]
3 legal criteria of consent	Legal characteristics; client is required to demonstrate ability in the following: Knowledge—basic recognition of the other person, relationship, and sexual activity in question. Intelligence—(rationality and understanding) aware of potential risks in the sexual relationship. Voluntariness—ability to resist or stop the sexual activity and identify willingness to continue.	[3,5-7,9, 10, 19, 26, 40, 89-93]
Ames and Samowitz instrument	Legal and clinical characteristics based on 3 legal criteria of consent; has 2 categories, A and B; consent determined by communication and behavior. Category B determines client consent ability based on their behavior showing the following: Voluntariness. Safety and avoidance of harm. No exploitation. No abuse. Ability to say no. Socially appropriate time and place.	[5,6,28,94]
Mental Capacity Act 2005	Legal characteristics; based in England and Wales; section 1 of the Act assumes the people have capacity to consent unless proven otherwise; knowledge and resources to aid the person’s decisions are encouraged; Includes rules for SDMs^b^. Consent requires the person to understand the following: Is there understanding of the decision that needs to be made and why? Does the individual understand the probable consequences when making the decision? Is the individual capable of understanding, remembering, deliberating, and using information that pertains to the decision? Is the individual able to communicate his or her decision in any way?	[66,95-97]
Lyden approach	Legal and clinical characteristics; endorses the 3 legal criteria of consent; encourages person-centered and integrated approaches; has important points for individualizing the assessment process, especially for communication. Has three general methods for determining the consent ability of a person with a cognitive disability, including the following: Review the relevant records (including info on reproductive ability and other disabilities). Create discussions, including those who know or work with the person being assessed. Conduct a personal interview to determine knowledge and voluntariness, supplemented with a mental status evaluation.	[5,26,89,92]
ABA/APA^c^ model	Legal and clinical characteristics; based on 3 legal criteria of consent, Lyden approach and Lichtenberg and Strzepek Instrument; expands on above models to include steps on how to enhance consent capacity and form comprehensive neuropsychological testing components; recommends LTC^d^ facilities to develop policies and procedures for sexual relations that are consistent with state statutes.	[10,22,26,88]
Vancouver Coastal Health Authority 2009	Clinical characteristics; downloadable manual. Provides recommendations for homecare staff and nurses such as the following: Respect the rights of persons with the capacity to consent to sexual activity. Do not reveal confidential specifics about the person’s sexual activity to those not directly involved in their care (including family members), without the person’s expressed consent, if the person has capacity. Remember that people who do not have capacity to consent to sex are still sexual beings with intimacy needs. Remember that not every person is heterosexual. Address one’s own attitudes and behavior toward older adults and general sexuality.	[16,98]

^a^MMSE: Mini-Mental State Exam.

^b^SDM: substitute decision maker.

^c^ABA/APA: American Bar Association and American Psychological Association.

^d^LTC: long-term care.

**Table 2:** The approaches used to determine and manage sexual consent abilities for people with cognitive disabilities.

Approach, type, and details					References
**Advance directive**					
	**Managing consent**				
		**Older adults with cognitive disabilities**			
			*Living wills* for the continuation or startup of relationships in advance.	[99,100]	
**Integrated approach**					
	**Determining consent**				
		**Cognitive disabilities**			
			I-team^a^ discussion, client assessments, enforcing client rights and education.	[88,92,97]	
			Reduce *all-or-none* SDM^b^ decision outcomes on client rights.	[83]	
		**Older adults with cognitive disabilities**			
			I-team, person-centered, interval checkups, and review policy with SDM.	[89,101]	
			I-team, person-centered, emphasis on client limits and their context.	[10,22]	
		**Inappropriate behavioral disabilities**			
			Client screening process, semistructured interview, and I-Team discussion.	[93]	
**Person-centered approach**					
	**Determining consent**				
		**Cognitive disabilities**			
			Holistic case-by-case, based on needs and policy, and client and staff education.	[4,23,29]	
		**Older adults with cognitive disabilities**			
			The 4 Ps: prioritize people, practice effectively, preserve safety, and promote trust.	[16]	
			Committee approach—staff, family, friends, residents, and client discussion.	[26,28]	
**Education**					
	**Managing consent**				
		**Cognitive disabilities**			
			Teach awareness of normal sex behavior to both clients and staff.	[27,34,95]	
			Client education checked by SCEA^c^, VABS^d^, or IQ tests.	[40,86]	
		**Developmental disabilities**			
			Consult certified sexuality educators or experts such as AASECT^e^ or OWL^f^.	[11]	
			Increase client sex-related knowledge, based on 3 legal criteria of consent.	[3]	
		**Older adults with cognitive disabilities**			
			Training for professionals and LGBTQ^g^ toolkits (info packages) for them.	[94]	
**Attitude**					
	**Managing consent**				
		**Cognitive disabilities**			
			Policy feminist disability theory, consent culture, and rely less on assessment.	[17,18,66,102]	
			Positive liberty, client proactive education, and attention to LGBTQ issues.	[48]	
			Social reframing. Recognize ability without facilitating pity.	[91]	
		**Older adults with cognitive disabilities**			
			Request and consult national resources to train teams for clientele.	[85]	
		**Inappropriate behavioral disabilities**			
			Psychological, social, and facility improvements over drugs. Staff education.	[41]	
**Functional capacity**					
	**Determining consent**				
		**Medical condition (stroke or comatose)**			
			Consent-Plus with committee input, MMSE^h^ (or similar), and interviews.	[19]	
		**Cognitive disabilities**			
			SSAS^i^ assessment, based on the 3 legal criteria of consent.	[90]	
			Focus on client act-specific action (not partner choice) based on MCA 2005^j^.	[96]	
			Adaptive capacity—correlate client’s other abilities to sexual consent.	[1]	
			Sex consent requires basic, consequential knowledge.	[9]	
		**Older adults with cognitive disabilities**			
			Assessments (MMSE and IQ), coupled with witness statements and context.	[103]	
		**People with psychiatric conditions (schizophrenia, personality)**			
			Communicate situational and internal understanding.	[84]	
**Support network**					
	**Both**				
		**Older adults with cognitive disabilities**			
			Cognition-plus. Determines consent, managed with family, staff, and SDM.	[81]	
**Contextual**					
	**Determining consent**				
		**Mild cognitive disabilities**			
			Consent assessment is kept the same among people and based on context.	[12]	

^a^I-Team: interdisciplinary team.

^b^SDM: substitute decision maker.

^c^SCEA: Sexual Consent and Education Assessment.

^d^VABS: Vineland Adaptive Behavior Scale (Interview Edition).

^e^AASECT: American Association of Sexuality Educators, Counselors, and Therapists.

^f^OWL: Our Whole Lives.

^g^LGBTQ: lesbian, gay, bisexual, transgender, transsexual, and queer.

^h^MMSE: Mini-Mental State Exam.

^i^SSAS: Social Sexual Awareness Scale.

^j^MCA 2005: Mental Capacity Act, 2005.

**Table 3:** Studies on sexual consent and education for people with cognitive disabilities.

Study type	Approach	Aim	Key findings	References
Qualitative	Integrated	Interview facility staff and residents to determine factors that increase risk of SDMs^a^ deciding *all-or-none* resolutions of resident consent capacity to sexual relationships instead of allowing partial expression.	Wording of legislation, lack of resources for SDMs and relational dynamics between them and staff increase risks of *all-or-none* decision outcomes. Recommends addressing these factors in integrated approach to reduce risk.	[83]
Qualitative	Attitude and education	Semistructured interview needs assessment of directors of nursing to identify challenges to sexual expression management in LTC^b^ setting.	Directors of nursing requested sexual expression to be addressed in a top-down manner, with national organizations’ support in resources and training.	[85]
Qualitative	Functional capacity	Interview facility staff and residents to determine key components of sexual consent.	Three key themes participants defined for consent: communication—includes all involved in sexual relationship either verbal or nonverbal, situational understanding—includes ability for all involved to interpret assent of partners, and internal understanding—includes personal understanding of desire for sexual relationship.	[84]
Qualitative	Education and attitude	Survey with vignettes to check residential staff ability to properly identify safe or unsafe sexual behaviors and respond accordingly.	Staff could generally identify the difference between abusive and safe sexual behavior. Increased age of staff correlated with less accuracy in identifying safe or unsafe behavior.	[34]
Qualitative	Functional capacity	Survey of APA^c^ to determine important criteria to determine key components of sexual consent.	Key themes defined for consent: basic sexual knowledge, knowledge of the consequences of sexual behavior, and aptitudes related to self-protection.	[9]
Quantitative	Education	Education intervention—*Living Your Life*, twice weekly, 45 minutes per session, 10-week total, to improve sexuality-related decision ability.	SCEA^d^ scale showed improved scores after education. Retention showed only slight decay after 6-month follow-up.	[86]
Quantitative	Education and functional capacity	Functional approach cohort study compared sexual consent ability of people living with cognitive disabilities to presumed normal people.	Some people with cognitive disabilities scored high on all measures, including the Sex-Ken-ID^e^. Recommended ongoing education instead of single inoculation model.	[40]
Quantitative	Functional capacity	Cross-sectional validity measure used SCEA to compare neuropsychological tests with IQ, adaptive behavioral age, and sex education on consent ability.	Neuropsychological test battery, especially those measuring executive measures, were found to be more accurate in predicting competency than IQ, adaptive behavior age, and sex education.	[45]

^a^SDM: substitute decision maker.

^b^LTC: long-term care.

^c^APA: American Psychological Association.

^d^SCEA: Sexual Consent and Education Assessment.

^e^Sex-Ken-ID: Sex Knowledge, Experience, and Needs Scale for People with Intellectual Disabilities.

### Themes Affecting Approaches for Determining Sexual Consent Capacity

Most reports (n*=*14) placed the 3 legal criteria of consent themes at the forefront of their approach to determine the sexual consent capacity of people living with cognitive disabilities. Other reports described adapted instruments, such as the Lichtenberg and Strzepek Instrument (n=4) or the Ames and Samowitz Instrument (n=4), which are approaches based on the 3 legal criteria of consent, however, with clinical considerations. The Lichtenberg and Strzepek Instrument mentions the use of an interdisciplinary team for the second part of its assessment process, which incorporated a team of professionals (eg, psychologist, psychiatrist, nurses, recreational therapists, dietitians, and aide staff) to analyze the information obtained by a psychologist or psychiatrist’s interview with the *patient* in the first part [87]. The Lyden approach, with person-centered and integrated considerations, has important points for individualizing the assessment process, especially for communication during the interview process. The American Bar Association and American Psychological Association (ABA/APA) model has a handbook, which is based on the 3 legal criteria of consent, Lyden approach, and Lichtenberg and Strzepek Instrument. It includes comprehensive neuropsychological testing components for determining consent capacity; however, it only summarizes the team-based aspects of determination and care plans [88].

With the 3 legal criteria of consent being based in the United States, some international reports described the Mental Capacity Act of 2005 as their main approach (n=4). The Mental Capacity Act of 2005 uses rules reminiscent of the 3 legal criteria of consent and contains the prefix assumption that a person has the default capacity to consent unless proven otherwise. The Mental Capacity Act of 2005 also contains prefix rules to ensure that knowledge and resources are available for assisting a person to make a consensual decision. Some reports introduced white papers and guides for assisting adult sexual health in LTC facilities (n=2), the most recommended guide being from the Vancouver Coastal Health Authority 2009. This guide provides important reminders to nurses and homecare staff regarding the rights of people under their care, while also recommending support to healthy sexual behavior by providing the means to do so (eg, provision of private spaces to reduce public sexual activity) [98].

### Approaches for Determining Sexual Consent Ability

#### Functional Capacity

Approaches endorsing the use of functional capacity have shifted away from diagnostic-based assessments (eg, IQ and mental age scores) of decision-making ability to alternative identifiers. There was an overall emphasis in the literature to rely less on mental assessment outcomes when determining the sexual decision-making capacity of people with cognitive disabilities [17,28,85,91,102]. The problem with mental assessments is the risk of social or cultural factors, concluding that people with cognitive disabilities are incompetent in society, which results in such people being placed in the *cloak of competence* [104]. Repeated assessment measures show mixed results for the same person, varying between having and not having a sexual consent capacity [3]. Thus, even if people with cognitive disabilities are able to demonstrate sexual consent capacity, social judgment may still suspect such people as being less than capable when compared with normative scores of intelligence and adaptive behavioral ability [91].

Despite the shortcomings of mental assessments, reports vouching for functional capacity approaches recommended to either expand the assessment’s ability to check for adaptive behavioral domains [1,19], featuring the use of neuropsychological assessments [88] or relegate such mental assessments to a supporting role [103]. Although a mental state assessment such as the MMSE may provide a basic idea on how well a person with cognitive disabilities makes rational decisions, it was recommended to consider other domains of functional capacity such as literacy skills, self-care skills, independent care ability, and physical upkeep [1]. A secondary diagnosis, including checks for both cognitive and functional ability, should be used when making a capacity judgment [103]. A report by Bogacki et al [90] introduced the Social Sexual Awareness Scale, a scale based on the 3 legal criteria of consent that checks a person’s knowledge for contextual and safety factors such as risk of disease, contraceptive use, age of partners, and the handling of unwanted advances [90]. Another recommendation was to assess decision-making capacity on act-specific decisions (eg, being able to make a decision on a sexual relationship, retain that decision, and have a rudimentary understanding of the sexual act) instead of theoretical ability [96]. One report suggested a holistic approach, complete with a progressive committee [19], supplemented with theories of Consensual-Minimalism and Consent-Plus by Wertheimer [105]. Consensual-Minimalism checks for the most straightforward sense of consent among those in a relationship, whereas Consent-Plus pertains to situations where consent is necessary in addition to other factors that are additionally required to make the sexual relationship permissible (eg, social, religious, and cultural factors) [105].

#### Person-Centered Approach

The person-centered approach is philosophically driven to promote ethical integrity when working with people to determine their consent capacity [4]. Discussions with the people themselves will enable a better understanding of both their sexuality and cognitive disabilities, which are essential for determining their preferences [15,99]. The person-centered approach needs to be flexible to accommodate the specific needs of each individual, promote their dignity and autonomy, and uncover potential contexts that could identify risks associated with sexual expression [16,18]. In terms of philosophical components throughout the literature, the person-centered approach was recommended to include the following (people living with cognitive disabilities, who are to be provided such services, will be referred to as *clients* in this list):

1. Open communication—this factor begins with individualizing the communication process with clients, following key components of the Lichtenberg and Strzepek Instrument, Ames and Samowitz Instrument, Lyden approach, ABA/APA model, and Vancouver Coastal Health Authority themes [5,6,87,98]. At the same time, given the personal biases and stigmas associated with sexuality, everyone including caregivers and staff should discuss such potential issues while planning to address them [23].
2. Committee approach—following open communication, this factor can *diffuse liability exposure and provide enhanced objectivity* [5]. If persistent evidence shows that a client’s consent ability has become compromised, any decisions involving their rights to sexual intimacy must involve discussions with family, friends, caregivers, and staff in a *multidisciplinary team* setting [16]. Such a committee can incorporate various perspectives to enhance a client’s autonomy, dignity, and rights to sexual expression in addition to determining areas and means for improving their potential shortcomings in the 3 legal criteria of consent themes [5,26]. Lay-witnesses are useful for determining other contextual information, such as the client’s adaptive capacity, in addition to determining potential SDMs if allowed [1,89].
3. Capacity assumed—a client’s sexual decision-making capacity needs to be assumed intact unless proven otherwise [4,83]. It is unethical and discriminatory to use assessments to prove that a client is incapable of demonstrating consent capacity [17]. A common-sense approach, with staff and peers observing the client’s interactions, nonverbal language, and social cues should instead be considered when determining their sexual decision-making capacity [4].
4. Withhold Bias—people’s attitudes, including those of staff members and caregivers, may inadvertently be against a client’s sexual preferences and deny them their rights to sexual expression [91]. It was strongly recommended for both clients and staff to receive education programs to discern differences between normative and unhealthy sexual behaviors in addition to reframing their perceptions regarding client sexual preferences and rights [3,23,29,34,88,92].
5. Tracking—a client’s sexual preference and decision-making ability is expected to change over time; thus, person-centered approaches should track a client’s progress, reconfirming that they retain the capacity to both understand and refuse a sexual interaction when necessary [23]. The impact of educational programs for enhancing a client’s sexual consent capacity should also be used and tracked over time [40]. Improving the client’s access to materials for practicing safe sex, such as condoms and contraceptives, should be continuously implemented [23]. Safeguards that limit forms of client sexual expression, such as restricting a relationship to kissing and touching only, requires continual monitoring by staff to ensure that such safeguards are not exceeded [5].

One report presented a system for nurses to use, which adheres to some of the previously mentioned philosophical components. The system comprises the 4 themes of the code, Professional Standards of Practice and Behavior for Nurses and Midwives [106]. The 4 themes are prioritize people, practice effectively, preserve safety, and promote professionalism and trust [16]. It includes considerations such as withholding bias, open communication, and the committee approach. A report by Wilkins [107] suggested an emphasis among a substituted judgment, best interest standard, or a mix of the two. The substituted judgment standard emphasizes the use of advanced directives, whereas the best interest standard emphasizes the balance between risks and benefits, allowing some restricted forms of client sexual expression if the potential benefits are worth the risks.

#### Integrated Approach

This is perhaps the most comprehensive approach in terms of providing a detailed care plan for determining sexual consent capacity in people with cognitive disabilities, while also discussing plans for enhancing consent capacity if necessary. The key features of the integrated approach include an interdisciplinary team discussion process, using aspects of a person-centered approach, complete with other holistic considerations. With the service user’s permission, the interdisciplinary team can be comprised an array of practitioners including physicians, occupational therapists, psychologists, social workers, nurses, and legal guardians of the person involved in the discussion [22]. The integrated approach is likely to encompass the following themes:

1. The 3 legal criteria of consent—for legally defining the terms of consent [7,10,22].
2. ABA/APA model—for blueprinting the overall assessment and care plan design process, endorsing person-centered considerations of the evaluated person’s sexual values, which endorses the Lichtenberg and Strzepek instruments for both functional capacity and ethical considerations [87,88].
3. Lyden approach—for individualizing the communication and assessment process, encouraging person-centered aspects to the approach [5,22,88].
4. Lichtenberg and Strzepek Instrument—assuming that the assessment aspect of the process is performed with an MMSE [87,88]; however, such assessments were designated as a supplementary role to determine where lacking areas of knowledge could be improved [48].

Friends and family of the evaluated person are encouraged to play a role in the discussion process [22]. Team input determines the restrictions of sexual expression, if any and medications to be prescribed, if any, while also noting contextual factors of the relationship, such as potential risks of coercion or abuse [22,92]. Overall, the integrated approach focuses on holistic contextual factors throughout the assessment process, including factors such as the person’s communication ability, access to privacy, informed consent ability, family involvement, religious beliefs, and social history [22,88].

#### Contextual Approach

The contextual approach was aimed at individuals with mild cognitive disabilities and has 2 components [12]. First, whenever a judicial system assesses the consent ability of an individual living with a cognitive disability, the ruling must meet the same standard as anyone else. Second, it is recommended that consent ability be focused on the situational context rather than on intellectual attributes. For example, an individual with a cognitive disability may show consent capability in a healthy relationship but not when their partner uses coercion or threats.

The first component becomes especially important when consent definitions require an understanding of tests involving the nature, consequences, and moral dimensions of sexual acts [12]. It is important to keep assessments among individuals the same, whether they have intellectual disabilities, because this focuses on social innerworkings within an intimate relationship [12]. This component also respects the capabilities of individuals with cognitive disabilities. The second component realizes that consent ability is affected by social constructs such as communication, social skills, and community support. A contextual approach reassures the balance of protection toward vulnerable persons while respecting their consensual rights to sexual relationships.

### Approaches for Managing Sexual Consent Ability

#### Education

There was a pattern in the reports explaining how education could improve the sexual decision-making ability of people living with cognitive disabilities. The pattern starts by mentioning the 3 legal criteria of consent components (knowledge, understanding, and voluntariness), followed by a defined set of basic skill checks to determine whether such people could address these components. Note that the 3 legal criteria of consent have a knowledge component, which defaults to being improved by sex education; however, its other components, such as understanding, may benefit from education as well [3,5,40,86]. The basic skill checks included areas that expanded upon the three legal criteria of consent [3,5,11,40]:

1. Knowledge of body parts and sexual relationships and acts.
2. Knowledge of consequences from sexual relationships.
3. Understanding of appropriate sexual behavior and context for it.
4. Understanding of the voluntary nature of a sexual relationship.
5. Ability to recognize abusive situations.
6. Ability to be assertive in such situations to reject unwanted advances.

The reports endorsing educational approaches described measures that check for these areas, such as the Sexual Consent and Education Assessment [86], Sex Knowledge, Experience and Needs Scale for People with Intellectual Disabilities [40], and Tool for the Assessment of Levels of Knowledge Sexuality and Consent [11]. For educational programs themselves, the recommendations were the *Living Your Life—The Sex Education and Personal Development Resource for Special Education Needs* [86,108] and comprehensible evidence-based programs with simple language [3]. It is important for the aforementioned assessments to be used only for the identification of gaps in a person’s knowledge of safe sexual acts, followed by providing educational programs to rectify such gaps if necessary [48,92].

In addition to people with cognitive disabilities, it was encouraged for staff in LTC facilities to receive education to better identify the difference between healthy and unhealthy sexual behaviors and how to resolve such situations accordingly [27,34]. Both families and LTC staff were recommended to receive education to better understand the rights to intimacy, sexuality, and privacy for people with cognitive disabilities [27]. It was suggested that staff use *LGBTQ toolkits*, which are manuals that describe ethical approaches when working with older female adults who have these sexual identities [94].

Criminal justice systems were encouraged to use education and training programs to increase the awareness of communication disorders, while also considering alternative communication platforms and multidisciplinary collaborations with relevant disciplines [109]. Children and people with communication disabilities are at a disadvantage when disclosing their experiences of sexual abuse to a criminal justice system, often because the system’s procedures may not be adapted to meet the needs of such people [109-111]. Sexual abuse cases showed improved outcomes when collaborative support was combined with communication awareness, such as for law enforcement and child protection services [111]. Improved access to sexual and gender-based violence education for vulnerable populations, such as refugees, was also recommended in addition to encouraging inclusion in community support programs [109].

#### Attitude

There were 3 articles that argued for both disability and feminist rights movements to overcome negative attitudes within communities [17,18,102]. Doyle [18] described how social construction theory for feminism relates to people with cognitive disabilities, defining it as culturally set norms, rights, and commitments that detail expectations on how people of differing statuses relate to one another [112]. Doyle [18] also explained that script theory is a form of construction theory that influences a person’s sexual behaviors by external and internal factors, defined as society’s *mutually shared conventions and norms* and personal motivations, respectively [18,113]. Script theory explains how the sexuality of a person with cognitive disabilities is potentially influenced by critical factors beyond just sex drive and instinct: it has learned behavior characteristics, influenced by social contexts, affecting how people express themselves [18].

Negative cultural attitudes, such as rape culture, should be countered by plans using lifelong sexuality education and policy change with *intermovement collaboration*, addressing the aforementioned internal and external factors [17]. A person with a cognitive disability could be incapable of demonstrating or understanding consent capacity, due to a lack of knowledge or having misaligned sexual scripts [18]; however, educational interventions may allow that person to reach capacity [5]. Note that a strict education approach emphasizes sexuality instruction to fill missing gaps in knowledge and understanding of consent criteria, although the attitude approach is often based on social construction theory, suggesting the use of education to reframe a person’s sexual scripts.

The attitude approach also discussed how a person with cognitive disabilities and their external factors such as government, legal systems, administration, practitioners, staff of LTC facilities, and family could be influenced. A study by Syme et al [85] determined that a proactive approach to policy development in LTCs was recommended, in addition to addressing negative staff and family attitudes. Addressing the lack of awareness of sexual expression in people with cognitive disabilities, making necessary environmental changes to ensure privacy, identifying staff or family attitudes, and tracking the person’s sexuality with recurrent assessments were the top areas to address in LTC facilities [85]. The study by Syme et al [85] found that all nursing directors (n*=*20) endorsed the use of sexuality education, with *many* endorsing attitude discussions with staff and family about aging sexuality, changing negative attitudes, increasing people’s awareness of their own attitudes, and emphasizing sexual health. A report by Victor [23] mentioned the use of staff attitudes about intimacy and dementia survey proposed by Kuhn [114] to measure staff or caregiver attitudes on this topic. A collaborative reform process was recommended to change legal terms (eg, mentally impaired to vulnerable or protected persons) when reframing government views that hold people to a disproportionately higher standard [92]. For committee approaches, members were tasked with reducing the risk of personal bias within the leading assessor, by providing a *balanced exchange of ideas in a competent and thorough manner*, throughout the consent capacity determination process [5].

#### Advanced Directives

Advanced directives are contingency plans that allow people to consent to specific sexual acts ahead of time or grant decision-making power to an SDM for an applicable future context [115]. The plan involves the person, who is legally capable of providing sexual consent at the time, the past self, to set up a contingency for an impending period when their sexual decision-making ability may become compromised, the future self. This compromised ability to provide sexual consent may occur owing to impending conditions from events such as dementia, stroke, or brain surgery. There are two types of advanced directives [115]: instructional directives and proxy directives. The instructional directives can be either permissive, allowing permissions to take place when they legally could not or restrictive, halting actions from happening when situations would normally favor them [115]. The proxy directive features the use of SDMs, either as a surrogate decision maker or the power of attorney [115].

A person’s ability to consent to sex can change over time, varying across situations in terms of capacity and sexual preference [3,5,11]. Unless legal exceptions have been established, it is illegal for consent to be given by someone else [7]. Advanced directives are noted for upholding a person’s core values and religious beliefs when resolving decisions involving sexual relationships, perhaps preserving the sexual preferences of a person living with a cognitive disability [77]; however, the drawback is that the person is *locked into certain conditions that may not coincide with the desires of the future self* [28]. The use of advanced directives in this manner is referred to as the substituted judgment standard [28]. One report presented the *Prior Consent Thesis*, a philosophy-driven argument that states that a competent person can give valid prior consent to a competent partner, in which consent could remain even after mental capacity in one person becomes compromised [100]. Sexual advance directives are not promises that could lead to the promiser being locked in servitude. The advance directive is not about owing a commitment; a person’s advance directive merely states that such an encounter be allowed if they token consent [100].

#### Support Network

Using the cognition-plus test, this approach contains three steps [81]: (1) check if the individual is capable of communicating a desire for an intimate relationship, (2) check if the individual is aware of the nature and consequences of sexual decisions, and (3) determine the adequacy of an individual’s decision-making support network.

If step 1 is unfulfilled, the test ends. Individuals who cannot determine an intimate relationship cannot qualify as sexual agents. If steps 1-2 are fulfilled, the individual is deemed to have consent capacity without the need for assistance. Step 3 becomes active only if step 2 is unfulfilled. The determination of an individual’s support network is contextual-based, guided by the fiduciary law [81]. The legal system would need to check if the support network is free from conflict of interest, while showing an understanding of the individual’s sexual preferences, with contingencies to protect the individual against consequences of sexual encounters, such as pregnancy or sexually transmitted disease [81]. The support network is expected to be different among individuals but may comprise the individual’s friends, family, institutional staff, and SDMs. SDMs are not recommended to act alone in a support network [81]. It is possible that people in the support network may have disagreements on the individual’s preferences, in which case these disagreements will need to be resolved in a civil manner before this approach is implemented [81]. The legal system should not intervene whenever a civil dispute occurs: its focus should only be on checking the support network’s adequacy [81]. It is important to note that support networks should not exist to make decisions for people with cognitive disabilities but only to assist them in achieving their decisions [81].

## Discussion

### Principal Findings

This review focused on the approaches used to determine and manage sexual consent abilities in people with cognitive disabilities, noting the recurring themes influencing how these approaches were implemented. The literature assumes that such people are capable of having the capacity to desire and consent to healthy intimate relationships; however, some situations may ignore or suppress these capabilities [5]. The movement of medicalized models to be enhanced by social support was also emphasized.

### Key Points When Determining Sexual Consent Ability

Review of the literature has established that determining consensual abilities requires a holistic approach, with individuals being considered in terms of their adaptive abilities, capacities, and human rights. An abridged description of such a holistic approach includes identification of the person’s sexual identity, beliefs and values, opinions from friends and family, medical records and clinical interviews (person-centered), neuropsychological testing, and functional capacity measures involving adaptive capability skill checks, followed preferably by an interdisciplinary discussion and action plan [88]. Although the 3 legal criteria of consent may appear as the starting point for defining a person’s consent capacity, sexual identity, beliefs, values, culture, and life history should be examined first to guide the consent-determining process [10]. This promotes the person-centered approach, especially if the assessed individual is of the LGBTQ community. The use of a committee during person-centered and integrated approaches must maintain a morality balance within the main reviewer; otherwise, the consent determination process can be skewed either in favor of or against the assessed individual [91]. Continual monitoring of an individual’s sexual preferences allows caregivers or service providers to offer empathetic maintenance over time, which becomes especially useful during cases of *fading identity*, owing to conditions such as dementia [23]. Educational programs focused specifically on these identified preferences can be provided to improve empathetic maintenance for such service providers.

The functional capacity report by Harris [1] provided examples of evidence for sexual consent ability in people with cognitive disabilities, including those demonstrating skills such as exercising *good judgment* and being able to identity their name and address correctly. However, the report by Harris [1] also admits that appraising these examples can be an *abstract exercise*, with some forms of evidence carrying a higher amount of probative weight than others, such as previous experience with sex education over IQ scores. A report by Thomas [84] provided elements of consent capacity definitions (communication, situational, and internal understanding), which were recommended to be presented to other residential mental health experts to create and improve the acceptable definition of consent ability. The most important consideration when using the functional capacity approach is to enforce that people with cognitive disabilities are not regulated differently than people without cognitive disabilities. If they should be denied legal right to sexual consent, the conclusion should only be drawn if the functional capacity appraisal process is performed on a truly equal basis for all [66].

Critical requirements of consent culture, which states that “people can have sex only when everyone agrees it is OK” [17], is met with scenarios where a person in a married relationship wants to have sex with their partner who has severe dementia, incapable of showing either signs of assent or refusal. Although it is important for all parties in the relationship to show the ability to refuse a sexual encounter at any point, which many people with cognitive disabilities will be incapable of doing [23], one proposed idea is to affiliate consensual sex with a continuation aspect, assuming consent from a previous loving relationship will remain even after someone loses the ability to consent afterward [100]. This continuation aspect of a sexual relationship with someone living with a cognitive disability is the *Prior Consent Thesis*, which deems current sexual relationships permissible, providing both people in the relationship gave consent to sex with each other before.

### Key Points When Managing and Enhancing Sexual Consent Ability

The key aspects to consider when managing and enhancing consensual ability in people with cognitive disabilities starts with attitude change. Some of the recurring attitudinal barriers identified in the literature include internal factors, such as those inflicting the individuals themselves as explained by script theory and external factors, peripheral to the individual affecting their consent ability and rights, examples being care providers, legal systems, family, friends, and supportive decision makers [18]. The attitude approach gives the impression of being the most important approach because of its ability to reframe either the internal or external factors’ view of sexual expression for people with cognitive disabilities.

For internal factors, script theory explained that people with cognitive disabilities may show unhealthy sexual behaviors because of unlearned scripts [18], which can be supplemented by education to fortify the knowledge and understanding prongs in the 3 legal criteria of consent theme. Script theory also explains that people may have intact sexual knowledge; however, *vital elements* pertaining to healthy attitudes can be misaligned, which may result in unhealthy behaviors directed at oneself or other people [18]. It has been reported that sexual knowledge alone does not always transfer to safer sexual behaviors [116]; thus, the importance of lived experiences on consensual ability emphasizes the importance of understanding the contextual reason why people with cognitive disabilities may consent to sex [18]. It is important to note that script theory does not explain inherent sexuality within individuals. Sometimes, unhealthy sexual behaviors stem from physical conditions, such as those from affected neurobiological areas in the brain within a person living with dementia [93].

External factors influencing sexual expression in people with cognitive disabilities include both formal and local situations. A report by Arstein-Kerslake and Flynn [102] provided details of a grassroots voice movement, using feminist disability theory to encourage a formal legal system to adopt vital changes to its interpretation of sexual rights for people with cognitive disabilities, further describing the drafting of its convention by a disabled people’s organization to reform the rights of such people. Perhaps these details may be enough to convince a similar rights movement to attempt their own reform process; however, the unique details pertaining to each convention’s attitudinal barriers will need to be reported in future literature for conventions to strategize and gain a greater level of confidence in attitude change. The more details a system can obtain for establishing attitude change, the louder a new convention can have a voice.

To check and address internal and external factors in the local situation, attitude checks using tools such as the staff attitudes about intimacy and dementia survey were recommended to give a general idea about potential staff, family, and caregiver biases toward sexual preferences in people with cognitive disabilities [23,93,114]. Publicly available guides, such as the Vancouver Coastal Health Authority Guide (2009), provide recommendations about components of staff education, SDMs, and family decision-making, including what to do in case decisions fall upon family members who do not support the client’s sexual activity. For situations with decision makers who do not agree with their clients’ sexual preferences, example guidelines include reiterating the legal obligations of such decision makers while reassuring them that they do not have to change their personal values—they only need to respect the legal rights of the client, especially after they have been given sex education [98].

The attitude approach is arguably the most important approach to consider, because all efforts to realize a person’s sexual consent ability can be lost, should the final decision fall onto someone who does not agree with the sexual preference in the first place. The comprehensive aspects of the integrated approach are not immune to this. The Hillman report [22] described an in-depth integrated approach that was used to assist with the sexual consent ability of a resident in a nursing home; however, the final outcome resulted in the family relocating the resident from there because of a difference in perceptions regarding sexual relationships. Using education to reframe staff and family attitudes is key because to quote one director of nursing from the study by Syme et al [85], “If the families don’t buy it, it’s gonna fail miserably.” The Boni-Saenz [81] report mentioned that support networks may fail because cognition-plus does not force its members to agree with their sexual preferences [81]. This suggests that both education and attitude approaches may ensure that consent plans are fulfilled properly. It was recommended for newer doctrines to reframe attitudes and counter stereotyping beliefs within those who serve people with cognitive disabilities, using laws to *exert positive expressive pressure on social norms* [81].

Advanced directives have evolved to a point where they may play a role in sexual decision-making. They are best established within third-party systems, especially those equipped to monitor their use, such as those in LTC settings [115]. Advanced directives may improve the decisional accuracy of institutional staff and loved ones who may act on behalf of the person living with a cognitive disability [117] or act as an *essential element* in a legal case against sexual assault [115]. The literature has revealed that the use of SDMs has been controversial. Although an SDM may appear qualified to make a decision on behalf of a person for a future sexual situation, it is possible the SDM may develop a conflict of interest, failing to act with loyalty in their proxy decision-making, resulting in potential *congruence problems* [118]. Having an advanced directive coupled with LTC facility monitoring or other such third-party settings reduces the risk of objective harm [115]. Further controversy stems from the disagreements among systems adopting the use of SDMs and the UNCRPD. The UNCRPD is against the use of SDMs, owing to the motion by Article 12 to establish equal rights for those living with cognitive disabilities [31,32]. The UNCRPD states that a lack of consensual capacity should not restrict a person’s ability to make a decision, insisting that said person’s *will and preferences* must be acknowledged [31,32]. Several experts have accused this interpretation as unrealistic [32,119-121]. Forgoing the use of SDMs would reduce civil commitment and potentially increase the risk of harm for individuals living with severe cognitive or psychotic disorders [32].

There are philosophical arguments that may prefer either the individual’s past or future self to take precedence over the final decision of the advanced directive. It is important for evidence of both the past and future self to show some form of communication to consent, be it verbal or nonverbal. If the past self had a contingency to consent to a sexual relationship and the future self showed a token of interest, such as overtly wanting to hold hands with someone they like, this may show an overlap in interests between the past and future selves. The noticeable overlap in consensual interests between past and future selves is known as the Consensus of Consents [115], which is the key to deciding whether such an advance directive is legally enforceable. Unfortunately, a person with a cognitive disability may show behavior that is less obvious in showing contemporaneous consent. It could be that some people living with dementia will have conditions so dire that they cannot communicate any form of consent at all. In these cases, the ability to express some form of definitive volition is required, either in the form of a verbal *yes* or some behavioral initiation of sexual expression [122]. With this volitional requirement, the voluntariness prong of the 3 legal criteria of consent demonstrates that sexual advance directives have both legal and medical domains, thus requiring dedicated supporters to have knowledge about consent capacity, mental conditions, and the very people living with cognitive disabilities themselves.

### Limitations

This review used a systematic method to identify approaches for determining and managing sexual consent capacity in people with cognitive disabilities. There was an emphasis on recognizing the patterns of themes, each influencing how consent-determining and enhancement programs were implemented. Although the literature on the subject may have a diverse array of ideas, acknowledging the views and rights of those who desire intimate relationships, this review emphasizes a convergent style to bring these ideas together. With this review’s emphasis on pattern recognition for noting recurring themes, there is a strong possibility that emergent ideas may have been downplayed or missed entirely. This review did not include ideas from conference papers, public opinions, or non–peer-reviewed articles. This may have shifted this review’s focus to a stronger understanding of already-established sexual consent themes; however, it could be that newer ideas may change these already-existing themes. Future research may provide emergent ideas with a greater consideration of the subject. In addition, although this review featured reports from both clinical and legal sources, this review predominantly used a clinical search protocol to locate the literature. The search process was not dedicated to the legal databases. Future research on this topic should be performed with a legal background, incorporating the necessary legal databases and journals.

### Conclusions

The desire to have an intimate relationship is one of the core elements of sexuality, which is part of what it is to be human. Healthy sexual relationships are driven by consent, which is commonly defined by people’s capability to demonstrate sexual knowledge, intelligence, and voluntariness. However, if a person with a cognitive disability has a compromised consent ability, the involved legal, clinical, or ethical systems must determine the balance between permitting and restricting sexual activity to reduce the risk of unhealthy or harmful sexual behavior. It is important for the attitudes of those involved in this process to be balanced; otherwise, the sexual rights of such assessed people could be moved either in favor or against them. The means for determining the sexual consent ability of people with cognitive disabilities include functional capacity and person-centered and integrated approaches. Management of consent ability includes education, attitude, and advanced directive approaches. These approaches seek the ideal outcome where person-centered considerations of those living with cognitive disabilities are understood and they themselves are involved in the process of personalizing the approaches used to facilitate healthy intimate relationships.

## Appendix

Multimedia Appendix 1 Search strategy.

## Abbreviations

ABA/APA: American Bar Association and American Psychological Association
LTC: long-term care
MMSE: Mini-Mental Status Exam
SDM: substitute decision maker
UNCRPD: United Nations Convention on the Rights of Persons with Disabilities
