# Supplementary material for: Approaches to Determine and Manage Sexual Consent Abilities for People With Cognitive Disabilities: Systematic Review
Source: Interact J Med Res. 2022 Feb 4;11(1):e28137. doi: 10.2196/28137 (PMC8857692; doi:10.2196/28137)

Appendix 1

Search strategy

Embase (OVID Interface) - 79 results
March 3, 2020

1. ((sex* or intima*) adj10 (consent or consensual)).mp.

2. (((intellectual* or mental* or cognitiv*) adj4 (impair* or disab* or deficit*)) or "long term care" or longterm-care or "nursing home*" or alzheimer* or dementia or autis* or Down* Syndrome).mp.

3. 1 and 2
4. limit 3 to conference abstracts
5. 3 not 4

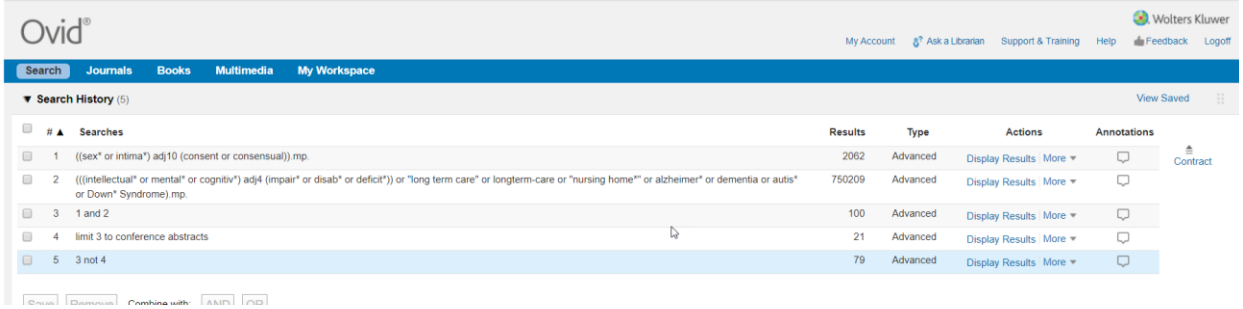

#	Searches	Results	Type	Actions	Annotations
1	((sex* or intima*) adj10 (consent or consensual)).mp.	2062	Advanced	Display Results More	
2	(((intellectual* or mental* or cognitiv*) adj4 (impair* or disab* or deficit*)) or "long term care" or longterm-care or "nursing home*" or alzheimer* or dementia or autis* or Down* Syndrome).mp.	750209	Advanced	Display Results More	
3	1 and 2	100	Advanced	Display Results More	
4	limit 3 to conference abstracts	21	Advanced	Display Results More	
5	3 not 4	79	Advanced	Display Results More	

Psycinfo (OVID Interface) - 83 results
March 3, 2020

1. ((sex* or intima*) adj10 (consent or consensual)).mp.

2. (((intellectual* or mental* or cognitiv*) adj4 (impair* or disab* or deficit*)) or "long term care" or longterm-care or "nursing home*" or alzheimer* or dementia or autis* or Down* Syndrome).mp.

3. 1 and 2

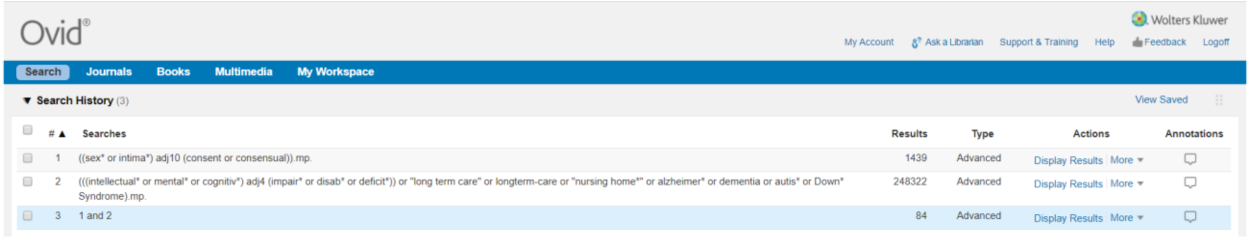

#	Searches	Results	Type	Actions	Annotations
1	((sex* or intima*) adj10 (consent or consensual)).mp.	1439	Advanced	Display Results More	
2	(((intellectual* or mental* or cognitiv*) adj4 (impair* or disab* or deficit*)) or "long term care" or longterm-care or "nursing home*" or alzheimer* or dementia or autis* or Down* Syndrome).mp.	248322	Advanced	Display Results More	
3	1 and 2	84	Advanced	Display Results More	

Scopus - 111
March 3, 2020
(TITLE-ABS-KEY (((sex* OR intima*) W/10 (consent OR consensual))) AND TITLE-ABS-KEY ((((intellectual* OR mental* OR cognitiv*) W/4 (impair* OR disab* OR deficit*)) OR "long term care" OR longterm-care OR "nursing home*" OR alzheimer* OR dementia OR autis* OR down*-syndrome)))

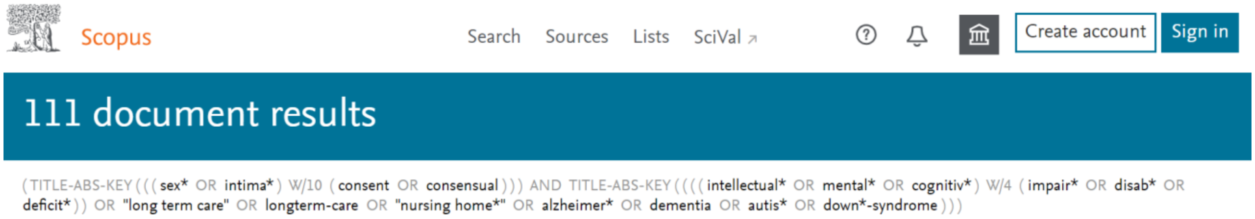

Ebscohost Interface databases:
MEDLINE (72)
CINAHL Plus with Full Text (60)
SocINDEX with Full Text (24)
Abstracts in Social Gerontology (10)
March 3, 2020

Deselect "Apply equivalent subjects"

(((sex* or intima*) N10 (consent or consensual))) AND ((((intellectual* or mental* or cognitiv*) N4 (impair* or disab* or deficit*)) OR "long term care" OR longterm-care OR "nursing home*" OR alzheimer* or dementia or autis* or Down* Syndrome))

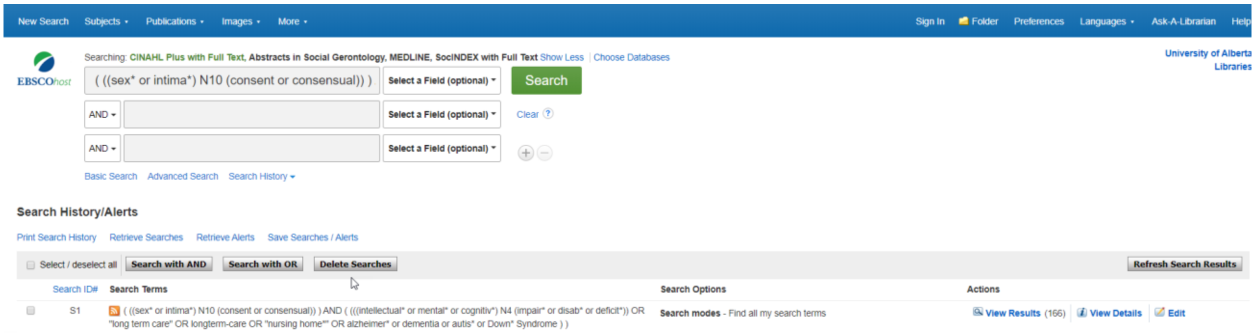

Supplement: Multimedia Appendix 1 [file ijmr_v11i1e28137_app1.pdf]
